# Adapting the adult social care outcomes toolkit (ASCOT) for use in care home quality monitoring: conceptual development and testing

**DOI:** 10.1186/s12913-015-0942-9

**Published:** 2015-08-04

**Authors:** Ann-Marie Towers, Jacquetta Holder, Nick Smith, Tanya Crowther, Ann Netten, Elizabeth Welch, Grace Collins

**Affiliations:** Personal Social Services Research Unit (PSSRU) George Allen Wing, Cornwallis Building, University of Kent, Canterbury, Kent CT2 7NF UK

**Keywords:** Quality monitoring, Care homes, Outcomes, ASCOT, Quality indicators

## Abstract

**Background:**

Alongside an increased policy and practice emphasis on outcomes in social care, English local authorities are now obliged to review quality at a service level to help in their new role of ensuring the development of diverse and high-quality care markets to meet the needs of all local people, including self-funders. The Adult Social Care Outcomes Toolkit (ASCOT) has been developed to measure the outcomes of social care for individuals in a variety of care settings. Local authorities have expressed an interest in exploring how the toolkit might be used for their own purposes, including quality monitoring. This study aimed to explore how the care homes version of the ASCOT toolkit might be adapted for use as a care home quality indicator and carry out some preliminary testing in two care homes for older adults.

**Methods:**

Consultations were carried out with professional and lay stakeholders, with an interest in using the tool or the ratings it would produce. These explored demand and potential uses for the measure and fed into the conceptual development. A draft toolkit and method for collecting the data was developed and the feasibility of using it for quality monitoring was tested with one local authority quality monitoring team in two homes for older adults.

**Results:**

Stakeholders expressed an interest in care home quality ratings based on residents’ outcomes but there were tensions around who might collect the data and how it might be shared. Feasibility testing suggested the measure had potential for use in quality monitoring but highlighted the importance of training in observational techniques and interviewing skills. The quality monitoring officers involved in the piloting recommended that relatives’ views be collected in advance of visits, through surveys not interviews.

**Conclusions:**

Following interest from another local authority, a larger evaluation of the measure for use in routine quality monitoring is planned. As part of this, the ratings made using this measure will be validated against the outcomes of individual residents and compared with the quality ratings of the regulator, the Care Quality Commission.

## Background

In England, as in many other countries, there has been increasing emphasis on the importance of considering the impact of services on outcomes and quality of life in health and social care policy, practice and research [1–4]. Despite the move towards community care, care homes remain one of the most expensive provisions of long-term care for frail older people in England [5], with local authorities spending £4,960 million on care homes for adults over the age of 65 in 2013–14 [5]. Ensuring that these services are providing good quality care is therefore high on the agenda of commissioners.

As part of an ambitious government agenda to change and improve adult social care, the 2014 Care Act [6] places responsibility on local authorities to ensure the quality of services they are commissioning and promote the well-being of people using them. The corresponding guidance for local authorities [7] specifically requires authorities to do this with reference to both the regulator’s minimum standards and the population outcomes outlined in the Adult Social Care Outcomes Framework (ASCOF) [8]. This emphasises the parallels being drawn between well-being, as outlined in the Act, and the quality of life of people with care and support needs; the overarching indicator for which is social-care related quality of life (SCRQoL), as measured by the Adult Social Care Outcomes Toolkit (ASCOT) ([8] p32).

Historically, local authorities have focused on monitoring the care provided to publicly-funded residents only. However, in a significant change of emphasis, local authorities now have a duty to facilitate local care markets to offer continuously improving, high quality services to all users, including those who fund their own care and do not rely on financial support from the local authority (self-funders) [9, 10, 7]. This wider remit lends itself to a more inclusive, home-level view of quality monitoring and may mean local authorities take a closer look at homes who care predominantly for self-funding residents.

There is very little literature providing a national picture of English local authorities’ quality assurance systems and audits. Think Local Act Personal (TLAP), a national partnership of organisations focused on transforming health and social care through personalisation, surveyed local authorities to try and find out more about their systems and the way in which their quality data is used. However, they only received 12 responses, representing 22 authorities; 11 individual authorities and one consortium of a further 11 [11]. Even with the consortium, given there are 152 local authorities with social services responsibilities in England, this represents only 14 % of local authorities. In total, seven reported conducting monitoring visits to assess care home quality, which included some observation of practice [11] and five maintain an online directory of some kind to provide information to the public about the services they accredit. Beyond the information made available by the regulator, historically, relatively little information about the quality of individual care homes in England has been made available to the public (see for a review [12]). Providers are generally considered opposed to the publication of individual provider performance data [13]. Although some local authorities have used information gathered during quality audits to indicate ‘preferred providers’ and used their own ratings or those of the regulator to incentivise providers through ‘payments by results’ [14, 15], this quality information is rarely shared with the public and so has typically not been able to support user choice.

There are an increasing number of ‘care ratings websites’ being provided by a variety of organisations, including the Social Care Institute for Excellence [16] and a care home sector led voluntary compact [17]. To aid choices about care and incentivise quality improvement through the provision of comparative information at provider level, the government launched a portal (www.nhs.uk) [2], which aims to draw together ‘high-quality’ information on the quality and effectiveness of individual care homes. Existing and planned quality marks, such as those of My Home Life [18], the Dementia Care and Support Compact [19] and NICE (National Institute for Clinical Excellence) quality standards [20] are intended to be included, along with the views of service users and their families, and specific information from providers on issues such as falls, staff training and turnover, medication errors and pressure sores [21].

A key source of information about the quality of health and social care is the regulator. Until 2010, star ratings (poor to excellent) were awarded to care homes by the then health and social care regulator, the Commission for Social Care inspection (CSCI). However, when CSCI was replaced by the Care Quality Commission (CQC), it withdrew the quality ratings and began a system of monitoring compliance against essential standards instead. Following the recommendations of reports into high profile abuse scandals [22, 23], a review of the value of quality ratings [24] and an independent review of how they carry out inspections [25], CQC carried out consultations on a new strategy for inspection [26]. This was followed six months later by another report, A Fresh Start [27], which outlined the feedback from the consultations and the proposed changes to the regulatory approach to collecting evidence, conducting inspections and judging quality, including the re-introduction of a quality ratings system (outstanding, good, requires improvement, inadequate).

In line with the Nuffield review of the value of quality ratings [24], CQC will now ask whether services are; safe, caring, effective, responsive and well-led and rated accordingly, with a view to; increasing accountability, aiding choice, improving performance, spotting failure, and reassuring the public [2, 28, 24]. CQC will gather evidence relating to these key areas through ‘intelligent monitoring’ [29], including evidence gathered by providers and others about the service. However, there is currently no way for providers, commissioners or researchers to reliably measure, evaluate and report quality of life outcomes at the provider/organisation level. As well as providing helpful information for CQC and addressing the emphasis placed on quality of life and well-being in the Care Act, recent research suggests such an indicator would be valued by the public, with relatives and carers identifying a measure of residents’ quality of life among their top three most useful indicators of care home quality [30].

The adult social care outcomes toolkit (ASCOT) currently offers a multi-method approach to establishing outcomes for individual care home residents based on eight domains of social care related quality of life (SCRQoL) (www.pssru.ac.uk/ascot). ASCOT was derived through a series of studies [31] and to date is the only measure focusing specifically on the areas of quality of life that can reasonably be attributed to social care services. The domains cover the basic (personal cleanliness and comfort, accommodation cleanliness and comfort, food and drink, and feeling safe) and higher order (social participation, occupation and control over daily life) aspects of SCRQoL, and there is also a domain to measure how the way the care and support is delivered impacts on service user’s self-esteem (dignity). ASCOT includes a care homes tool (CH3) which uses a multi-method approach (observation and individual interviews) to score the social care related quality of life (SCRQoL) of individual care home residents based on these domains (www.pssru.ac.uk/ascot).

The multi-method approach was developed due to the challenges of gathering self-report information from care home residents. Around two-thirds of care home residents in the UK have dementia [32, 33] and engaging people with cognitive impairment through surveys poses many challenges [34]. Observations have long been used as an ethnographic method of research in care homes [35] and can be particularly helpful when researchers are interested in the daily routines and interpersonal communications of residents and staff [36, 37]. As noted by Luff et al. [35], “while self-report scales and questionnaires are traditionally ‘quick and easy’ forms of data collection, this may not be the case when working with people living in care homes” (p.25), owing to the high levels of physical and cognitive frailty [38]. Furthermore, there is evidence that it is inappropriate to rely solely on the kind of self-report information collected through surveys/questionnaires when making judgements about the quality of a service [39, 40, 31].

The ASCOT toolkit, and in particular the care home interview and observation instruments, are cited as data sources for local data collection about quality measures identified by NICE for care homes for older people [41]. However, mixed-methods approaches to data collection are more time-consuming and resource intensive than self-completion surveys or interviews alone. This is justified and indeed appropriate when the goal is to measure the outcomes of individuals lacking the capacity to participate in other ways but does not lend itself to a ‘whole home’ approach. At the home level, individual ratings would need to be aggregated to either an average score for the home or reported at the domain level, indicating the distribution of outcomes in each. This kind of information is very sensitive to changes in the current population of residents, however, and as such may be better used by providers to profile residents and identify unmet needs and potential training issues. It is also a resource-intensive method of collecting data that relatively few would be able to undertake as part of their routine quality monitoring activities, let alone keep up to date. A home level (rather than individual level) measure may be able to fill the gap for a reliable outcomes-based approach to quality monitoring, assurance and improvement [42], and if made available to the public has potential to help people compare and choose an individual care home. However, before a new measure is developed it is important that the purposes are clear and the measure viewed as useful to potential users.

This paper presents the results of an exploratory study that sought to develop a new measure of care home quality, based on residents’ quality of life outcomes. We describe the consultations with stakeholders, outline how these fed into the conceptual development of the measure and end by reviewing the feedback from the feasibility testing in one local authority quality monitoring team.

## Methods

Three broad interlinked activities were carried out: consultations with stakeholders, tool development and feasibility testing with a quality monitoring team.

Key professional and lay stakeholders’ views were sought through a variety of methods: a one-day workshop, face-to-face interviews and focus groups. Each set out to identify views about potential use of the measure and associated methodological issues and to gather feedback on how the ASCOT domains and definitions might be adapted to work at a whole home level.

Professionals were invited to the workshop using opportunistic sampling of those who were already aware of and interested in ASCOT and had signed up to the ASCOT mailing list. This included; local authorities, care providers and their representatives, academics and voluntary organisations. We also purposively invited stakeholders that did not attend the workshop to take part in face-to-face meetings. During the workshop, small group consultation sessions, led by different members of the research team, were tape recorded and transcribed for later analysis. During the face-to-face meetings, participants did not wish to be tape recorded, so the research team took detailed notes instead.

Potential lay users of care home quality information were invited to take part in focus groups to complement the consultations with professionals. Ethical approval for this phase of the study was granted by the national Social Care Research Ethics Committee (SCREC) in June 2013. The lay groups aimed to include (1) relatives and carers of older people living in care homes, (2) relatives and carers of older people who have experience of social care services and support and (3) adults aged between 45 and 75 who may have to help arrange residential care for an older relative in the future, but as yet have no experience of choosing a care home. They were recruited through existing local groups in one local authority. Three local voluntary sector carer organisations agreed to help recruit groups 1 and 2. A University run database of members of the public willing to be research participants was accessed to recruit participants for group 3, however to address low response rates an advertisement was also posted on an online University website.

Following the consultation phase, the research team undertook a review of the findings and drew out the main messages for the development of the draft measure. Sometimes different stakeholders had different priorities and views of the proposed measure and these were discussed and reviewed on an iterative basis throughout the conceptual development phase. Using the ASCOT care homes toolkit as a starting point, and drawing on previous work undertaken for one local authority’s quality monitoring team, we began the conceptual and descriptive adaptation of the quality of life domain headings, descriptions and ratings system. We also drafted some provisional guidance and wrote training materials for the final phase.

In the final phase we explored the feasibility of quality monitoring (QM) officers using the new tool as part of their monitoring visits. This stage of the research was granted ethical approval from the national Social Care Research Ethics Committee (SCREC) in November 2013. We sent an email via the ASCOT mailing list asking for one local authority to pilot the draft measure in two homes for older people. Three local authorities expressed an interest and we recruited the one able to work within the time frames of the project. QM officers were trained to use the draft measure and then, working in pairs, they spent a day in each home collecting outcomes-focused data through: a 2–hour period of structured observation in communal areas (including lunch) and semi-structured interviews with staff, residents and family members, if available. The day after the visit, they each individually rated the home they had visited drawing on the evidence collected and the guidance and training we had given them. A face-to-face debrief meeting was held within a week of the visits to gather feedback on the data collection processes and ratings-system and explore whether they had disagreed about any of the ratings, and if so, why? We also explored the face-validity of the measure by asking the team to reflect on whether their final ratings gave an accurate depiction of their own views of the homes, drawing on their professional experience as quality monitoring officers.

All participants gave informed consent to participate in the research. Consent for the consultation phase was given verbally by professional stakeholders who voluntarily agreed to take part in interviews or attend the workshop. For everyone else, including the quality monitoring team piloting the draft toolkit, consent was given in writing.

## Results

### Consultation phase

#### Sample

Seventeen adults (13 women and 4 men) with and without current caring responsibilities took part in the focus groups in 2013. 16 provided further demographic information. Of those, all stated their ethnicity as White. Two participants were in the 45–54 age bracket, eight were aged 55–64, five were aged 65–75 and one participant was in the bracket of 75–84 years. 12 were married/living in a civil partnership, one was widowed, two were cohabiting/living as married and one was divorced. 11 identified themselves as carers and 15 had experience of knowing/helping someone move into a care home. Despite our attempts to recruit people who might potentially use care home quality information in the future but who currently had no experience of doing so, all but two of the participants had experience of helping a parent, parent-in-law or spouse choose a care home. Most reported experiences of choosing permanent placements, although two had looked for a short-term placement.

The workshop attendees (*N* = 28) included care home providers, local authority staff, representatives from membership body for the voluntary care sector, Skills for Care, HealthWatch, NICE, SCIE and professionals involved in education and training in the sector (e.g. around end of life care). We also interviewed representatives from a membership body for the nursing home sector (*N* = 2), a local authority unable to attend the workshop (*N* = 1) and the health and social care regulator (CQC) (*N* = 2). Professional stakeholders came from several regions of England including; the South East, London, the Midlands, North East and the North West.

It was clear from the consultation phase that who collects the data entirely affects how the data might be used and in particular, whether or not it would be helpful for the public. Focus group participants said they would find the information helpful when choosing a home for themselves or their family members and believed it would serve to drive up quality by focusing homes on outcomes for residents. However, they also noted that they would only consider the ratings trustworthy if provided by an independent organisation or if they represented the views of relatives and those who had stayed in the home. Ratings based on first-hand experience were considered more reliable than judgements made by health and social care professionals, who they felt might have ‘an agenda’ based on making cost-savings. There are currently other mechanisms in place for gaining the views of residents and their families, including; user satisfaction surveys carried out by providers and local authorities and the Your Care Rating survey developed by Ipsos MORI with the National Care Forum and Care England [43]. Indeed, the ASCOT is included in the user experience surveys sent out by local authorities each year. However, there is evidence to suggest that online quality information might not be that well utilised when choosing homes [30] and that surveys do not usually represent the views and experiences of the most impaired, often relying on the views of representatives or ‘proxies’, which are known to be different from the service users themselves [44, 45].

Professional stakeholders suggested that the consumer champion, Healthwatch, might be able to collect the data and make the ratings available to the public. Healthwatch is made up of local organisations based in each of the 152 local authorities in England (www.healthwatch.co.uk) and is commissioned by, but independent to, those local authorities. Healthwatch has statutory powers, including being able to enter health and social care services to conduct quality reviews but relies heavily on volunteers to operationalise its objectives. Nevertheless, at the time of writing, some authorities have already commissioned local Healthwatch organisations to carry out their own ‘enter and view’ visits from a quality monitoring perspective (e.g. Healthwatch Kent http://www.healthwatchkent.co.uk/projects). However, the success of this approach not only depends upon the skills, training and capacity of Healthwatch staff and volunteers but also the number of homes they inspect and their ability to keep ratings up-to-date. Recency of ratings and frequency of data collection were themes that arose during the focus groups with members of the public, with participants agreeing that ratings should be updated every six months to be considered reliable. Unless ratings are available on all homes their potential to aid user choice is limited. Homes without ratings, as well as those who have been rated poorly or as requiring improvement, may justifiably feel they are at a disadvantage and users may experience frustration when they cannot directly compare across short-listed homes.

Unsurprisingly, given that this project was prompted by interest from local authorities, a key use of the measure was for quality monitoring and improvement and workshop participants noted that its focus on residents’ outcomes fitted well with wider regulatory and policy changes. Compared with Healthwatch, local authority quality monitoring teams are likely to have greater coverage in terms of the number of homes they audit. At the very least they should collect information about the homes they fund placements in. However, much like the lay stakeholders, they said their ratings should not be made publicly available. There seemed to be two interrelated reasons for this: firstly, many local authorities wanted to work in partnership with providers to improve quality and publishing ratings was seen as potentially damaging to positive relationships (echoing the view that providers are generally against the publication of what is considered ‘performance data’); and secondly, there were concerns about local authorities’ capacity to keep ratings up-to-date (which is one of the reasons providers are against such ratings being published). This raises the question of who, other than the providers themselves, would have the resources to keep ratings up to date in a way that would be considered fair to providers and helpful to the public. This is also a challenge for CQC [46], especially in the current financial climate.

### Development of the draft measure

#### Background information

ASCOT has eight domains of social care-related quality of life (see Table 1), with one item per domain [31]. Domain descriptions are purposively broad so as to be relevant to all adults using social care services, including younger adults, those living in the community and those in paid or voluntary work. The full toolkit and associated guidance and scoring systems can be viewed here www.pssru.ac.uk/ascot. Self-completion and interview versions of ASCOT have four response options per item. In these versions, the top two states make a distinction between no needs and the ideal situation and are phrased in the language of capabilities [47]: whether or not people are able to achieve their desired situation [31].

The existing care homes toolkit for individual residents (CH3) has three response options per item worded in the language of ‘functionings’ (no needs, some needs and high needs), based on the principle that nobody should maintain such a poor level of functioning in any domain that there are health implications if their needs are not met [47]. As outcomes in the care homes toolkit are ‘rated’ by observers to enable the inclusion of people with cognitive impairment, a domain rating of no needs is the best outcome that can be given. No needs indicates that the person has no unmet needs in that area of their life; some needs means that they have some unmet needs and it is having a negative effect on their quality of life and high needs are distinguished from some needs by being severe or numerous enough to have physical or mental health implications. For example, in the case of food and drink, people who do not have meals at times they would like or choice over what to eat would have some needs; those who were getting an inadequate diet or insufficient liquids would have high needs.

#### The measure

It was clear from the consultation phase and the interest from local authorities that potential users wanted this measure to operate as a driver for continuous quality improvement. As such, the home level toolkit needed to go beyond simply recognising when residents’ needs are met. ASCOT has potential to do this at the home-level through an adaptation of the capabilities approach and by extending the existing three-level ratings system to four, in line with the self-completion and interview tools. As homes are increasingly striving to deliver person-centred care [48, 49] we decided to conceptualise the top level in these terms. Each domain will be rated according to one of four possible outcomes states, shown in Table 2. The best outcome is conceptualised in terms of the delivery of personalised care and support.

**Table 1:** The ASCOT domains

Domain title	Domain description
Control over daily life	The service user can choose what to do and when to do it, having control over his/her daily life and activities
Personal cleanliness and comfort	The service user feels he/she is personally clean and comfortable and looks presentable or, at best, is dressed and groomed in a way that reflects his/her personal preferences
Food and drink	The service user feels he/she has a nutritious, varied and culturally appropriate diet with enough food and drink he/she enjoys at regular and timely intervals
Personal safety	The service user feels safe and secure. This means being free from fear of abuse, falling or other physical harm and fear of being attacked or robbed
Social participation and involvement	The service user is content with their social situation, where social situation is taken to mean the sustenance of meaningful relationships with friends and family, and feeling involved or part of a community, should this be important to the service user
Occupation	The service user is sufficiently occupied in a range of meaningful activities whether it be formal employment, unpaid work, caring for others or leisure activities
Accommodation cleanliness and comfort	The service user feels their home environment, including all the rooms, is clean and comfortable
Dignity	The negative and positive psychological impact of support and care on the service user’s personal sense of significance

Taken from http://www.pssru.ac.uk/ascot/domains.php

**Table 2:** CH4-HL ratings states from best to worst

Domain title	Domain description
Best outcome	Residents have outstanding quality of life in this area. All residents are being cared for and supported in a consistently personalised way with their wishes and feelings being taken into account.
	Residents have good quality of life in this area. All residents are cared for and supported in a way that meets their needs.
	Residents have an inadequate quality of life in this area. Some residents are not having their needs met and there are enough issues to affect their quality of life although there is no immediate risk to their health.
Worst outcome	Residents have a poor quality of life in this area. Residents’ needs are not being met and their physical or psychological health is being put at risk because there are so many issues or because the issues are so serious.

**Table 3:** Domain titles, subheadings and definitions for draft measure CH4-HL

CH4-HL Domains	Definitions
Accommodation	Residents live in a clean and comfortable home and like how it looks and feels. Bedrooms and shared areas are well designed, easy to get around and meet residents’ health and social care needs.
Living in a clean and comfortable home	
Personal cleanliness and comfort	Residents are clean and comfortable. They are dressed in ways that meet their individual needs and wishes.
Being clean and presentable	
Food and drink	Residents eat and drink well. They get a balanced and varied diet, including food they like and need.
Eating and drinking well	
Personal safety	Residents feel safe and free from fear of physical and psychological harm and are supported to manage risks.
Feeling safe and free from fear	
Being sociable	Residents spend time socialising with people they like and taking part in social activities. Close relationships with family, friends (from inside and outside the home), carers and people from the wider community are supported.
Spending time with people, being sociable.	
Being occupied	Residents spend time doing things they like, value and enjoy on their own or with others. They are supported in continuing activities that they have been involved in the past.
Having things to do, being occupied	
Choice and control over daily life	Residents have choice and control over their daily life. They feel they ‘have a say’ in their care, daily routine and activities and that their views are respected.
Having choices, feeling in control	
Dignity	Residents are treated with compassion, dignity and respect. Staff think about what they say and how they say it and consider the feelings of residents when giving care and support.
Being treated with dignity and respect by staff	

How these outcomes states are described will depend on how the measure is used and by whom and will require further work and testing than was possible in this study. Initial plans had been to label them outstanding, good, inadequate and poor but during the consultation phase some local authorities and providers indicated that these labels were not helpful and were laden with negative connotations. For quality improvement purposes, some preferred ‘grades’ (A, B, C, D) and others suggested a traffic light system (green through to red), although how this might be conceptualised at four levels requires further testing. CQC have adopted a traffic light system in their new quality ratings, with the top two quality ratings both being green but being distinguished by shape; outstanding is a green star and good is a green circle (http://www.cqc.org.uk/content/care-homes). A similar approach might work well for these ratings but some professional consultees felt that this toolkit needed to be very different to any approach taken by the regulator, to avoid confusion. Currently, ratings are made at the domain level and although they could be arbitrarily assigned a value, which could be summed to create a raw ‘score’, the validity of such a score would also require further testing. As the aim of this study was to create a draft toolkit and explore its feasibility as a care home quality indicator, not develop a final toolkit, this was considered acceptable.

Unlike the individual level outcomes states, the definitions of the new home-level states need to account for variation in quality of life between residents in a home. To do this, we included quantifiers such as ‘all’ and ‘some’ in the top level definitions and provided additional guidance for those planning to use the toolkit to rate homes. For example, if any residents are experiencing poor or inadequate quality of life for a particular domain, the home cannot be rated as outstanding in that domain. For quality improvement purposes, homes would receive a rating, contextualised with evidence collected from observation and interviews. Professional stakeholders wanted the wording of this measure to be written from the perspective of the person using the service and be tailored specifically for residential care. To avoid losing comparability with the original ASCOT measures, we accommodated this by introducing subheadings for each domain. Stakeholders also expressed a preference for the measure to avoid language that might infer care home residents are passive recipients (e.g. *residents* receive), as this was considered contrary to the current policy emphasis of placing service users ‘in control’. See Table 3 for the adapted CH4-HL domain headings and definitions.

The domains themselves were considered relevant to a care home quality indicator, with lay stakeholders spontaneously mentioning control over daily life, food and drink, occupation, social participation, safety and accommodation when asked what the proposed measure should include. There was considerable discussion amongst all stakeholders around the meaning and focus of the safety domain and what it added to the judgements made by CQC and other safeguarding systems. As ASCOT is rooted in the measurement of quality of life, it seemed appropriate for CH4-HL to focus primarily on how residents’ *feel* and be worded accordingly. Lay stakeholders also discussed including an indicator of staff compassion and empathy, linking well to the outcome referred to as ‘dignity’ in ASCOT, which is conceptualised in terms of the impact of the way staff treat you on your self-esteem. This domain also offers an explicit opportunity to evaluate relationships between staff and residents, with evidence of good outcomes coming from the nature, tone and warmth of communication in the home and feeding into final ratings. As relationship-centred care is being increasingly advocated in terms of best-practice in care homes [50], this is an important consideration of care home quality.

#### Draft methodology and guidance

CH4-HL has been adapted from the existing care homes toolkit, CH3, which used a mixed-methods approach to data collection, including; structured observations and interviews with residents, staff and family members, where possible. It takes approximately one day to collect CH3 data for every five care home residents [51], which is very resource-intensive, especially in large homes. Feedback from local authorities was that for CH4-HL to fit with existing quality monitoring visits and activities, raters should be able to collect all the information required to make the ratings in one day.

Primarily the toolkit is based on the ‘enter and view’ model [52] and as such places emphasis on structured observation of residents in communal areas. However, it is also important that residents, staff and family members have the opportunity to give their opinions as much as possible. Consequently, the draft guidance recommended that raters work in pairs and between them:

- Conduct a 2 h structured observation
- Interview at least five residents (2–3 each)
- Interview up to five staff members (2–3 each)
- Speak to family member and visitors, if available
- Interview the home manager

CH4-HL interviews were semi-structured around the eight domains to gather evidence on what life is like for residents in those domains. Ideally, residents would be interviewed first, followed by a period of observation, including the midday meal. This would then raise issues or queries that could be followed up with staff and the home manager in the afternoon. As recruitment of family members was likely to be opportunistic, our guidance recommended trying to speak to family members and visitors when the opportunity arose, rather than being prescriptive. The feasibility testing aimed to examine whether it was possible to collect all of this information in one day and whether teams felt it provided them with enough details to be able to make the ratings.

#### Feasibility testing

Five quality monitoring (QM) officers in one local authority were trained to use the measure as part of a routine visit. Working in pairs, four used the draft measure in two care homes for older adults. The characteristics of these homes are shown in Table 4. After their visits, each QM officer made their own independent ratings of the home they had visited before talking through their ratings with their colleague and identifying differences in opinion and why these might have occurred. In accordance with their preferences, the team piloted a grading system for ratings, with A being the best and D being the worst. Care homes were given a grade for each domain and a written explanation for that rating alongside. These were discussed with the home managers with a view to agreeing action points for quality improvement. The research team returned to the local authority approximately two weeks later to discuss their experiences and the feasibility of using the toolkit as a care home quality indicator in the future.

**Table 4:** Characteristics of homes involved in feasibility testing

	Home 1	Home 2
Type of home	Older adults without nursing	Older adults without nursing
Including dementia?	Yes	Yes
Capacity	28	29
Occupancy	27	27

#### Feedback from the quality monitoring team

Overall, the team felt that “*the day went well”* (QM2) but they reported struggling to achieve the desired number of interviews with staff and were unable to interview any relatives. Table 5 provides information about the homes and summarises how many interviews the teams managed to complete during their day. There were two main barriers to completing more interviews: interview length and the availability (or lack thereof) of staff and relatives. Upon discussion, it became clear that a more feasible approach would be to use the observations and resident interviews as a foundation for follow-up questions with staff, rather than complete interviews, and to target these around the issues identified. There was also discussion about sending ASCOT questionnaires to family members in advance of the visits, asking for their view of their relative’s quality of life that way. Training in observational methods and interviewing was highlighted as being particularly key. Teams found they sometimes struggled to stand out of the way (but in positions from which they could easily see what was happening) and found that some residents found their presence a concern because they were not clear (or lacked the capacity to understand) why they were there:

**Table 5:** Summary of data collected during the visits

	Home 1	Home 2
Manager present?	Yes	No-called away urgently
No. QM officers	2	2
Staff interviews^a^	2	1
Resident interviews^a^	4	4
Relative interviews^a^	0	0

^**a**^Aiming for 5 staff and *at least* 5 resident interviews per home. We included relative interviews but knew these were going to be opportunistic and difficult to achieve

*“There was a particular lady who said, “Oh, are you writing about me?” And she was saying, “Is that a bad report?””(QM2)*

This highlights the skilled nature of the work and suggests the measure should only be used by those who have had training in observational techniques to reduce the impact of observer bias effects. Although many quality monitoring officers have experience of ‘enter and view’ visits, some authorities still rely on paper-based monitoring and would require greater input to ensure staff are competent and confident in this approach. As such, it will be important for us to develop training materials alongside developing and testing this toolkit further in the future.

In terms of coverage, feedback about the domains was very positive: *“the domains cover the elements of what’s important for a home”* (QM1). Despite some anxiety about making the ratings without looking at care plans, they recognised that there was an intention to move away from paper-based monitoring and that spending all their time on care plans and policies was not the answer either:

*“you can have the documentation that’s brilliant but what you see in practice doesn’t reflect that…. and what makes a difference to them [residents] on a day to day basis is the interaction and that experience.” (QM2)*

In a sense, their concerns echoed what had already been picked up in the workshop and survey, in that much of their anxiety was around culpability and reliability. To collect all the data, the team had divided the interviews between them and sometimes seen or heard different things during their observations. Consequently, they found it essential to discuss the evidence before making their ratings. In terms of feasibility, this is important, suggesting that teams need to collect the data in pairs and then share information or allow one person two days to conduct a visit. Despite these challenges, however, they felt that the guidance led them to rate the home in a way that reflected their own feelings of what life was like for residents:

*“I felt that if I’d have gone in that home and done a quality monitoring visit, the normal visit, I’d have said, “That’s a good home,” which is where that came out with the toolkit” (*QM3)

A strength of using the ASCOT toolkit was that it provided an outcomes-focused, not process-focused, framework on which to base their visits/observations and transparent criteria against which to make their ratings. It will therefore be important to test the reliability of these ratings as part of a future pilot study.

#### Feedback from the homes

The QM team asked homes for their views on the data collection process and final ratings. Unfortunately, in one home the manager was called away from the home during the pilot testing and then felt unable to comment on the process and ratings. In the second home, staff felt the day was not disrupted in any way by their visit and the home manager said that the:

*“assessment/ report is fair and [it] will help me to address areas that raise some concerns so improvements can be made to the service we deliver to our residents” (Home Manager 1)*

After the visit, the home manager drew on the evidence gathered by the QM team to respond to the ratings in each domain. Although the home manager and the QM team did not always agree, having the evidence to support the ratings allowed them to enter into a dialogue about this and understand why those ratings had been given and agree actions for improvement, which is key for quality improvement and mutual respect and understanding between professionals in the sector.

## Discussion

This paper has explored the demand for a new care home quality measure based on residents’ outcomes and presented early development and feasibility testing. Feedback from the consultations with stakeholders and preliminary testing in one local authority indicates that there is a use for this measure, especially in local authority quality monitoring teams, and that the ASCOT domains work at the care home level. However, it also highlighted the skilled nature of collecting data about quality of life through structured observations and interviews and the importance of thorough information gathering to inform ratings. The observational element of the draft toolkit worked well but training would be required to ensure a consistent approach. If used in quality monitoring, the interviews require further work to fit with the time constraints of those collecting the data and ensure adequate information is collected from a variety of sources. Alternative modes of engagement, including postal questionnaires for family members, should be explored.

The potential for this measure to aid user choice arose several times during the consultation phase and reflects a wider political and cultural shift towards encouraging the public to adopt a consumer-approach to long-term care [53, 54]. Certainly previous research and the result of our own consultation indicates that the public would value a quality indicator based on residents’ outcomes [30] but this raises the issue of who would be responsible for collecting the data and making it public. Professional stakeholders suggested Healthwatch might be best placed to fulfil this role and this might be something than individual authorities and local Healthwatch teams might explore in the future. However, for such information to be truly of use to prospective residents and their families, ratings would need to be available on all homes in their area. With around 10, 087 homes for older people in England [55], this is not a small task and would require substantial resources, even with the use of trained volunteers. Furthermore, research evidence suggests the information may not be widely used by the public. The decision to move into a care home is often made at a time of crisis and constrained by the availability of places [56] and greater weight is often given to ‘word of mouth’ or the reviews of people who know the services [15, 57]. The previous regulator, the Commission for Social Care Inspection (CSCI), found that less than 1 % of social care users said they used the previous star ratings when making a decision about which home to move to [15].

Our own consultations with members of the public suggested a preference for information about the quality of homes to be grounded in the views of residents and their families. This is in line with the recent increase in ‘care ratings websites’ [see 30 for a review]. On its website, Your Care Rating states that it aims to: give care home residents a voice; promote continuous quality improvement and provide an “authoritative source of information for existing and prospective customers” (http://www.yourcarerating.org/about-us/). However, providers have to opt-in to the survey and in 2013 it was only sent out to 1, 123 homes, or approximately 11 % of care homes for older adults in England [43]. Thus, it is unlikely that the data held on this website will be used to help prospective users find a home. Furthermore, although participants are asked about important aspects of their quality of life (e.g. having visitors when they want, having their own possessions around them, taking part in activities), unlike ASCOT, the survey does not measure the outcomes of social care, which was something the public said they would find relevant and helpful. It is this outcomes-focused approach that makes the measure different from anything else currently available, including the ratings made by the care regulator. Although CQC’s decision to re-introduce quality ratings goes beyond only inspecting compliance, it is notable that the Care Act guidance for local authorities suggests that they use the “definition that underpin the CQC’s fundamental standards of care as a minimum” (p.44) but consider the outcomes included in the ASCOF when promoting quality [7].

Clearly a measure of care home quality based on ASCOT has the potential to be used in different ways by different stakeholders. However, without better information sharing between organisations, it might not be possible for the measure to do all of the things highlighted by stakeholders in this study. As Warmington [58] notes, there needs to be a collective accountability for the quality of care and better information sharing is a key part of this. For example, a key tension for this measure is whether, and how, ratings are made available to the public to aid user choice. For good coverage, consistency and authority, local authorities appear best placed and most interested in using the toolkit to carry out ‘enter and view’ visits. However, feasibility testing indicated they may not have the resources required to collect the experiences of relatives and visitors, which the public seem to value particularly highly. Perhaps there is scope for partnership work between local Healthwatch and quality monitoring teams, with Healthwatch collecting information about relatives’ views of the CH4-HL domains and the local authority conducting the monitoring visits? As local authorities were reluctant to make their final ratings available, but there was a clear desire for such information from members of the public, perhaps there is scope for only the results of the potential survey of relatives to be made publicly available to inform user choice? Under the Care Act (2014) authorities are required to provide prospective users, including self-funders, with information about the homes in their area. Information such as this could be shared upon such enquiries being made, thus avoiding the need for relatives and frail older people to ‘data mine’ themselves for reliable, relevant and current information.

## Conclusions

This study came about because local authorities expressed an interest in using the Adult Social Care Outcomes Toolkit in quality monitoring. However, it was a small feasibility study and our samples were not representative of the wider population. For example, we only managed to recruit people of white, British ethnicity for our focus groups and so have been unable to explore whether there are differences in the perspectives of other ethnic groups. This would be an interesting topic for further research.

Our aim was to explore the wider demand for such a measure and examine how it might be used and by whom. Since carrying out the feasibility testing, another local authority has approached us with a view to piloting the draft measure in their routine quality monitoring visits. We plan to evaluate this and examine how it relates to the ratings given by the CQC and the outcomes of individual residents living in the homes, with a view to validating the measure and scoring system and making it available for use by researchers, providers and local authorities in the future.

## Abbreviations

ASCOT: Adult social care outcomes toolkit
CSCI: Commission for social care inspection
CQC: Care quality commission
NICE: National Institute for Clinical Excellence
QM: Quality monitoring
SCIE: Social Care Institute of Excellence
SCRQoL: Social care related quality of life
TLAP: Think Local Act Personal
